# Red root rot alters root-zone microbial communities and enzyme activities in *Hevea brasiliensis*

**DOI:** 10.3389/fmicb.2026.1726470

**Published:** 2026-04-02

**Authors:** Chunping He, Chunxin Zhai, He Wu, Shibei Tan, Zhenhua Li, Yu Zhang, Ying Lu, Weihuai Wu, Yanqiong Liang, Kexian Yi

**Affiliations:** 1 Key Laboratory of Integrated Pest Management on Tropical Crops, Ministry of Agriculture and Rural Affairs, Environment and Plant Protection Institute, Chinese Academy of Tropical Agricultural Science, Haikou, China; 2 College of Plant Protection, Nanjing Agricultural University, Nanjing, China; 3 School of Tropical Agriculture and Forestry, Hainan University, Haikou, China; 4 Hainan Rubber Industry Group Co., Ltd., Haikou, China; 5 Sanya Research Institute of Chinese Academy of Tropical Agricultural Sciences, Sanya, China; 6 Hainan Key Laboratory for Detection and Control of Tropical Agricultural Pests, Haikou, China

**Keywords:** enzyme activity, *Hevea brasiliensis*, microbial diversity, pathogenic fungi, physicochemical properties, red root rot, rhizosphere microorganisms

## Abstract

**Introduction:**

The rubber tree (*Hevea brasiliensis*) is an important industrial raw material and strategic resource in China. Red root rot, caused by the pathogenic fungus *Ganoderma pseudoferreum*, is the most severe root disease and poses a serious threat to rubber production. Understanding the differences and correlations in rhizosphere soil microbial communities, environmental factors, and enzyme activities between healthy rubber trees and those infected with red root rot is of great significance for the green prevention and control of rubber tree root diseases and the regulation of soil microecology.

**Methods:**

In this study, Illumina MiSeq high-throughput sequencing technology was used to analyze the differences in the structure and composition of rhizosphere soil microbial communities between healthy rubber trees and those infected with red root rot. Combined with soil physicochemical properties and enzyme activity indicators, the relationships between microbial ecological characteristics (such as soil nutrients and soil enzyme activities) and the occurrence of red root rot were explored.

**Results:**

According to the results, the occurrence of red root rot increased the richness and diversity of bacterial and fungal populations in the rhizosphere soil. Fungal community composition has a greater impact on plant disease occurrence: the abundance of Actinobacteria in the rhizosphere soils of diseased plants is significantly lower than that of healthy plants, while the abundance of Ascomycota is significantly higher. Notable genus-level changes revealed a dramatic fungal community shift. The abundance of beneficial genus Termitomyces plummeted from 37.50% in healthy soils to 0.026% in diseased soils. Concurrently, *Hygrocybe*, *Peniophora*, unclassified *Archaeorhizomycetes*, and *Ganoderma* in diseased soils were significantly increased by 82.13%, 98.59%, 69.74%, and 97.87% compared with healthy soils, respectively. Diseased soils exhibited significantly higher pH, soil water content (SWC), total nitrogen (TN), invertase (INV), and catalase (CAT) activities, but lower nitrate nitrogen (NO₃-N) and acid phosphatase (ACP) activities. In addition, the study found that soil pH, TN, SWC, available phosphorus (AP), CAT, and INV were positively correlated with the relative abundances of *Ganoderma* and *Archaeorhizomyces*, while NO₃-N content and AP were positively correlated with the relative abundance of Termitomyces.

**Discussion:**

In summary, red root rot alters root-zone microbial communities and enzyme activities in *Hevea brasiliensis*, thereby clarifying the correlations between soil environmental factors and key microbial taxa (particularly *Ganoderma* spp. and rhizosphere bacteria). This study provides a crucial theoretical basis for advancing our understanding of the disease’s occurrence mechanism and microecological underpinnings, as well as for formulating targeted ecological management strategies for its control.

## 1 Introduction

Rubber trees (*Hevea brasiliensis* Muell. Arg.), a perennial deciduous tree belonging to the genus *Hevea* in the family Euphorbiaceae, are the primary source of natural rubber worldwide. Owing to their high elasticity, insulation properties, and wear resistance, they find extensive applications in aerospace, medical and healthcare, electrical and electronic industries, as well as the automotive sector (Shi et al., 2021). Beyond its role as an irreplaceable industrial feedstock and strategic commodity (He and Huang, 1987), *H. brasiliensis* also performs essential ecological functions such as carbon sequestration, soil and water conservation, microclimate modulation, and biodiversity preservation, thus emerging as a vital tree species integrating economic and ecological values in tropical areas (Singh et al., 2021). China is a major producer of both natural rubber and rubber products. Currently, its rubber planting area exceeds 11.3 million *mu* (approximately 1.7 million acres), with an annual output of 800,000 tons, accounting for about 6% of the global total. Ranking third and fourth in the world, respectively (Rong, 2023). Yunnan and Hainan provinces are the two main rubber-producing regions in China, contributing approximately 49.14 and 47.82% of the national planting area (Liu R. J. et al., 2022). However, China’s self-sufficiency rate in natural rubber is less than 15% (Ai and Huang, 2023), underscoring the crucial importance of ensure a secure supply of this resource.

In recent years, alongside the development of the natural rubber industry, root rot diseases in rubber trees have grown increasingly severe. This escalation can be attributed to factors such as long-term continuous cultivation, low economic returns, extensive management practices, and climate variability. These diseases have resulted in large-scale declines in latex production and even tree mortality, significantly undermining both the yield and quality of natural rubber. Among these, red root rot caused by infection with *Ganoderma pseudoferreum*, stands out as one of the most devastating soil-borne diseases affecting rubber trees. Red root rot is characterized by its high latency, strong infectivity, rapid transmission, and widespread occurrence, making it an extremely destructive pathogen. It often triggers secondary infestations, such as by bark beetles (*Scolytidae*), and if left untreated, the mortality rate can reach 100% (Bai et al., 2008). Due to its high resistance to control measures and the severe economic losses it incurs, this disease poses a major challenge to rubber plantation management. Currently, conventional control measures for red root rot primarily rely on chemical fungicides (e.g., tridemorph) for root irrigation and trenching to isolate infected roots. While these methods have shown moderate efficacy in mitigating the disease, they suffer from significant drawbacks, including high chemicals and labor costs, which hinder large-scale implementation. Given these limitations, biocontrol technologies have emerged as a promising alternative due to their environmentally friendliness. Notably, endophytic antagonistic bacteria (e.g., *Bacillus subtilis*) isolated from rubber trees have demonstrated significant suppressive effects against red root rot (Liang et al., 2022, 2023). However, in practice, the antagonistic effects of these beneficial bacteria against pathogens often fail to manifest as fully under field conditions as they do in controlled laboratory settings. This discrepancy arises from complex interactions involving the pathogen, host plant species, indigenous microbial communities, and environmental variables (Maraha et al., 2004; Mazzola and Freilich, 2017). Key challenges include the inability of introduced biocontrol strains to effectively compete with native microorganisms for resources, or unfavorable soil conditions that hinder their survival and colonization, ultimately compromising disease control efficacy. This underscores the need for integrated management strategies that account for multiple influencing factors. Crucially, understanding the relationship between microbial community structure and environmental variables may provide theoretical support for improved disease control (Bulluck and Ristaino, 2002). Therefore, elucidating the dynamic changes in rhizosphere soil microbial communities and enzyme activities during the progression of rubber tree root rot holds significant practical value for both red root disease management and rubber tree quality enhancement.

Red root rot primarily infects rubber trees through root contact with infected or dead root debris and mycelia in the soil (Li and Luo, 2007). As a critical interface between plants and soil, the rhizosphere plays a vital role in nutrient uptake and stress resistance (Li et al., 2025). Microorganisms, as key component of soil ecosystems, are essential for soil nutrient cycling, organic matter transformation, and energy metabolism (Pratscher et al., 2011; Kluge et al., 2015; Petersen et al., 2012). Their sensitivity to environmental changes makes them effective early indicators of soil quality and ecological function. Furthermore, the abundance and activity of rhizosphere microorganisms are closely linked to plant diseases dynamics (Arancon et al., 2004). Currently, the use of high-throughput sequencing technologies to characterize changes in soil microbial community diversity at the molecular level has become a research focus. Root rot pathogenesis is complex, with its occurrence closely associated with microbial community dysbiosis and deterioration of the soil microecological environment. Scholars have shown that there are significant differences in the community structures of bacterial and fungal in the rhizosphere soil between plants affected by diseases such as honeysuckle root rot (Li et al., 2025), flue-cured tobacco bacterial wilt (Wei et al., 2025), white peony root rot (Zhang et al., 2024), wheat head blight (Wang et al., 2024), Sanqi (*Panax notoginseng*) root rot (Wang et al., 2023), Coptis root rot (Tang et al., 2022), American ginseng root rot (Jiang et al., 2018; Yu et al., 2018), apple root rot (Yang et al., 2020), and cruciferous clubroot (Wu et al., 2020) and their healthy counterparts, with marked disparities in population diversity and richness.

Early research on rubber tree root diseases predominantly emphasized pathogen control agents and antagonistic microorganisms in the soil, overlooking the characteristic microorganisms (bacteria and fungi) of the entire microbial community, their interactions with soil properties, and their role in the onset of root rot. Consequently, a systematic investigation is warranted to examine root rot pathogenesis, shifts in soil microbial community diversity, and enzyme activities dynamics during rubber trees cultivation. This entails an in-depth analysis of the microbial community fluctuations between healthy and diseased soils, soil chemical properties, and the interplay between these factors and enzyme activities changes. Identifying the dominant contributors to red root rot in rubber trees within this region is crucial for advancing our understanding of its occurrence and dissemination, as well as for developing targeted prevention and control strategies. From the perspective of the rhizosphere soil microecology of rubber trees, this study focuses on the rhizosphere soils of both red root rot and healthy rubber trees. This work analyze the alterations in the microbial community structure within the rhizosphere soil, elucidate the relationships among soil microbial communities, rhizosphere environmental factors, and enzyme activities, and uncover the interaction mechanisms between soil microorganisms and environmental conditions in the context of red root rot in Qiongzhong, Hainan.

## 2 Materials and methods

### 2.1 Sample site and sampling collection

*Hevea brasiliensis* cultivar “PR107” was cultured in 1998 at Hongxing Team, Xinjin Farm, Qiongzhong County (19°13′53”N, 109°50′21″E), Hainan Province, China. The main climatic conditions of Qiongzhong County are as follows: tropical monsoon climate, annual mean temperature, 22 °C; annual average relative humidity, 80%; annual average precipitation, 2,300 mm; annual sunshine duration, 1700 h; altitude, 200–350 m; the predominant soil type, laterite soil. The planting density ranges from 30 to 33 trees per mu (approximately 666.7 m^2^), with a spacing arrangement of 2.5 m × 8 m. In November 2019, we conducted a field survey in this area and observed a 10% incidence of red root rot (caused by *Ganoderma pseudoferreum*) in the rubber trees at Hongxing Team.

Based on the incidence of red root rot in the experimental area, we selected three healthy rubber tree plots and three plots affected by red root rot for soil sample collection. Diseased plants were selected based on clear rubber tree root rot symptoms, including yellowing and stunted leaves of the aboveground parts and necrotic rotting of the belowground roots. Healthy plants were selected that were separated by at least four plants from diseased trees, characterized by vigorous growth with no disease symptoms observed in adjacent plants. Each plot measured 20 m × 20 m.

A cross-shaped sampling pattern was employed to randomly collect root-zone soil from two trees per plot (healthy rubber trees designated as ‘J’, diseased trees as ‘C’). Soil samples were obtained using a 7-cm diameter soil auger within a 1-meter radius from the trunk. Four auger cores (0–20 cm depth) were extracted per tree and composited after removing litter and gravel. Samples were sealed in bags labeled ‘C’ or ‘J’ and stored in an ice box, and transport to the laboratory within 12 h. Six red root rot affected soils (labeled C1-C6) and six healthy root-zone soils (labeled J1-J6) were collected.

The collected soil samples were thoroughly mixed, and sieved through a 2-mm mesh. These soils samples were split into three sections: one part of fresh soil samples were used to determine soil physicochemical properties including nitrate-nitrogen (NO₃-N) and ammonium-nitrogen (NH₄^+^-N); another part was subjected to basic physicochemical analyses and soil enzyme activities determinates following air drying; the remaining samples were kept at −80 °C for total DNA extraction.

### 2.2 Soil physicochemical properties and enzyme activities

The analysis of basic physicochemical properties of air-dried soil was performed assessing five key indicators: soil organic matter (SOM), total nitrogen (TN), available phosphorus (AP), available potassium (AK), and pH value. Measured using a pH meter (FE28-Standard, Mettler Toledo, Switzerland) with a water: soil ratio of 2.5:1 (v/w) according to the agricultural industry standard of the People’s Republic of China (NY/T 1377–2007) (Wang et al., 2007). Soil total nitrogen (TN) was detected by Kjeldahl nitrogen analyzer (Hanon K9840, Shandong, China). Soil available potassium (AK) was measured by the ammonium acetate extraction-flame photometry method (Lu, 1999). Soil available phosphorus (AP) and soil organic matter (SOM) were analyzed using soil physicochemical determination reagent kits produced by Suzhou Comin Biotechnology Co., Ltd. The activities of five key enzymes involved in the cycles of carbon (C), nitrogen (N), and phosphorus (P) were tested: *Urease* (URE), *Catalase* (CAT), *Polyphenol oxidase* (PPO), *Acid phosphatase* (ACP), and *β-Fructosidase* (INV). The activities of these five soil hydrolases were determined using assay kits manufactured by Suzhou Kemin Biotechnology Co., Ltd., China. For fresh soil, the content of ammonium nitrogen (NH₄^+^-N) and nitrate nitrogen (NO₃-N) were determined using analytical kits produced by Suzhou Comin Biotechnology Co., Ltd., China.

### 2.3 Soil microbiome diversity analysis

To compare the root-zone soils microbial community structure between healthy and red root rot of *H. brasiliensis*, we conducted the following analyses: Total DNA was extracted from 0.5 g homogenized soil samples using the E.Z.N.A.® Soil DNA Kit (Omega Bio-tek, GA, USA). Using diluted genomic DNA (20 ng/μL) as template, we performed PCR amplification targeting: the bacterial 16S rRNA V3-V4 region with primers 515F/806R (Caporaso et al., 2011), and the fungal ITS region with primers ITS1F/ITS2R (Zinger et al., 2009). The PCR reaction mix was 20 μL, consisted of 1 μL DNA template, 0.4 μL each of forward and reverse primers, 10 μL 2 × Taq PCR StarMix and 8.2 μL ddH₂O. The PCR amplification procedure involved initial denaturation at 95 °C for 5 min, followed by 35 cycles of denaturing at 95 °C for 30s, annealing at 55 °C for 30s, and extension at 72 °C for 60s, with a final single extension at 72 °C for 10 min, and subsequent cooling at 4 °C. The PCR products were purified using VAHTS™ DNA Clean Beads (Vazyme Biotech Co., Ltd., China), quantified with Qubit 3.0, and verified by 1.5% agarose gel electrophoresis. Afterward, the DNA was sequenced by Sangon Biotech Co., Ltd. (Shanghai, China) using an Illumina MiSeq PE300 platform.

### 2.4 Statistical analysis

Raw sequences underwent primer/adapter trimming and barcode-based demultiplexing in QIIME 1.9.0 (Caporaso et al., 2010) with stringent quality control (Q-score ≥20, minimum length 200 bp). Chimeric sequences were detected *de novo* and removed using USEARCH v11.0.667. High-quality reads were clustered into operational taxonomic units (OTUs) at 97% similarity threshold via the UPARSE (Edgar, 2013) algorithm. Taxonomic assignment was performed using the RDP Classifier v2.13 (Wang et al., 2007) against the Greengenes database (v13_8) with an 80% confidence cutoff.

Data were statistically processed using Microsoft Excel 2010 (Microsoft, Redmond, WA, USA) and JMP 10 (SAS Institute, Cary, NC, USA), with graphical presentations created in Origin 2019 (OriginLab, Northampton, MA, USA). Using IBM SPSS Statistics 25.0 (IBM Corp., Armonk, NY, USA), independent samples t-tests identified significant differences (*p* < 0.05) in soil physicochemical properties, enzyme activities, and microbial indices. Pearson correlation analysis was used to examine relationships between soil microbial community composition and both soil physicochemical properties and enzyme activities. The microbial community’s alpha diversity was quantified using three indices: Chao1 (richness), Shannon (diversity), and Simpson (evenness), while beta diversity was assessed through principal coordinate analysis (PCoA) of Unifrac-based dissimilarity matrices, implemented in MOTHUR v1.44.0 (Schloss et al., 2009).

## 3 Results

### 3.1 Soil physicochemical properties and enzyme activities

As shown in Table 1, there were significant alterations in root-zone soil properties of red root rot of *H. brasiliensis* compared to healthy trees (*p* < 0.05). Diseased trees exhibited 3.43% higher pH, 11.29% greater water content (SWC), and 9.31% increased total nitrogen (NT). Conversely, nitrate nitrogen (NO₃-N) content showed a 58.71% reduction in infected trees. No significant differences were detected in available phosphorus (AP), available potassium (AK), organic matter (SOM), or ammonium nitrogen (NH_4_^+^-N) levels between healthy and diseased specimens. Red root rot significantly altered enzyme activities in the root-zone soil of *H. brasiliensis* (*p* < 0.05). Compared with healthy plants, the non-rhizosphere soil, red root rot resulted in 23.01% higher catalase (CAT) and 15.19% elevated sucrase (INV) activities, while acid phosphatase (ACP) activity decreased by 4.5%. Urease (URE) and polyphenol oxidase (PPO) activities remained unaffected. These findings suggest that red root rot occurrence correlates with specific soil parameter changes (pH, SWC, TN and NO₃-N) and induces metabolic disturbances affecting energy metabolism, carbon cycling, and phosphorus conversion efficiency.

**Table 1:** Changes physicochemical properties and enzyme activities in root-zone soil of healthy and red root rot plants of rubber trees.

Index	J	C
pH	4.22 ± 0.05b	4.37 ± 0.04a
SWC (%)	17.13 ± 0.33b	19.31 ± 0.77a
TN (mg/kg)	736.17 ± 1.01b	811.79 ± 3.45a
NO_3_-N (mg/kg)	7.29 ± 2.25a	3.01 ± 0.64b
NH4^+^-N (mg/kg)	1.20 ± 0.45a	0.85 ± 0.17a
AP (mg/kg)	1.82 ± 0.27a	2.16 ± 0.55a
AK (mg/kg)	38.58 ± 1.10a	39.60 ± 3.76a
SOM (%)	0.96 ± 0.11a	1.07 ± 0.26a
URE (μg/d/g)	526.47 ± 8.96a	508.93 ± 7.02a
CAT (μmol/d/g)	6.02 ± 0.43b	7.82 ± 0.36a
INV (mg/d/g)	5.47 ± 0.14b	6.45 ± 0.15a
ACP (μmol/d/g)	18.64 ± 0.39a	17.80 ± 0.34b
PPO (mg/d/g)	5.18 ± 0.16a	5.14 ± 0.05a

J and C represent healthy and red root rot-affected root-zone soils, respectively. Values followed by different letters within the columns are significantly different according to independent samples *t*-tests (*p* < 0.05). The same as below.

### 3.2 Microbial gene abundance and *α* diversity in the soil microbiome

The α-diversity indices effectively reflect the abundance and diversity of microbial communities. As shown in Table 2, the bacterial gene copy number in the root-zone soil of healthy rubber trees was significantly higher (16.53% increase) than that in the root-zone soil of diseased plants (*p* < 0.05). However, the bacterial community richness (Chao1 index) was significantly lower in healthy plants compared to diseased plants root-zone soil (*p* < 0.05). Other bacterial diversity parameters including observed OTUs, Shannon index (species diversity), and Simpson index (evenness) showed slightly lower values or no significant differences in healthy plants compared to diseased soil samples (*p* > 0.05). For fungal communities, the gene copy number in the root-zone soil of diseased plants was significantly reduced by 52.63% compared to healthy plants (*p* < 0.05). In contrast, the fungal Chao1 index (richness), OTUs, and Shannon index were significantly higher in diseased plants, while the Simpson index was significantly lower than in healthy rubber tree root-zone soil (*p* < 0.05).

**Table 2:** The microbial gene abundance and *α*-diversity index in root-zone soil of healthy and red root rot plants of rubber trees.

Classification	Soil sample	α-diversity index				
		Gene copy number(×10^8^ copies/ g)	OTUs	Chao1	Shannon	Simpson
Bacteria	J	5.14 ± 1.22a	6116.67 ± 272.69a	9172.60 ± 325.46b	6.57 ± 0.07a	0.005 ± 0.001a
	C	4.29 ± 1.15b	6622.67 ± 229.78a	10056.03 ± 304.69a	6.71 ± 0.07a	0.004 ± 0.001a
Fungus	J	0.38 ± 0.22a	1465.67 ± 18.48b	1708.89 ± 33.81b	3.96 ± 0.16b	0.14 ± 0.02a
	C	0.18 ± 0.13b	1627.00 ± 44.84a	1928.41 ± 11.78a	4.91 ± 0.13a	0.03 ± 0.00b

### 3.3 Structural differences in soil microbial communities

**Figure 1:**
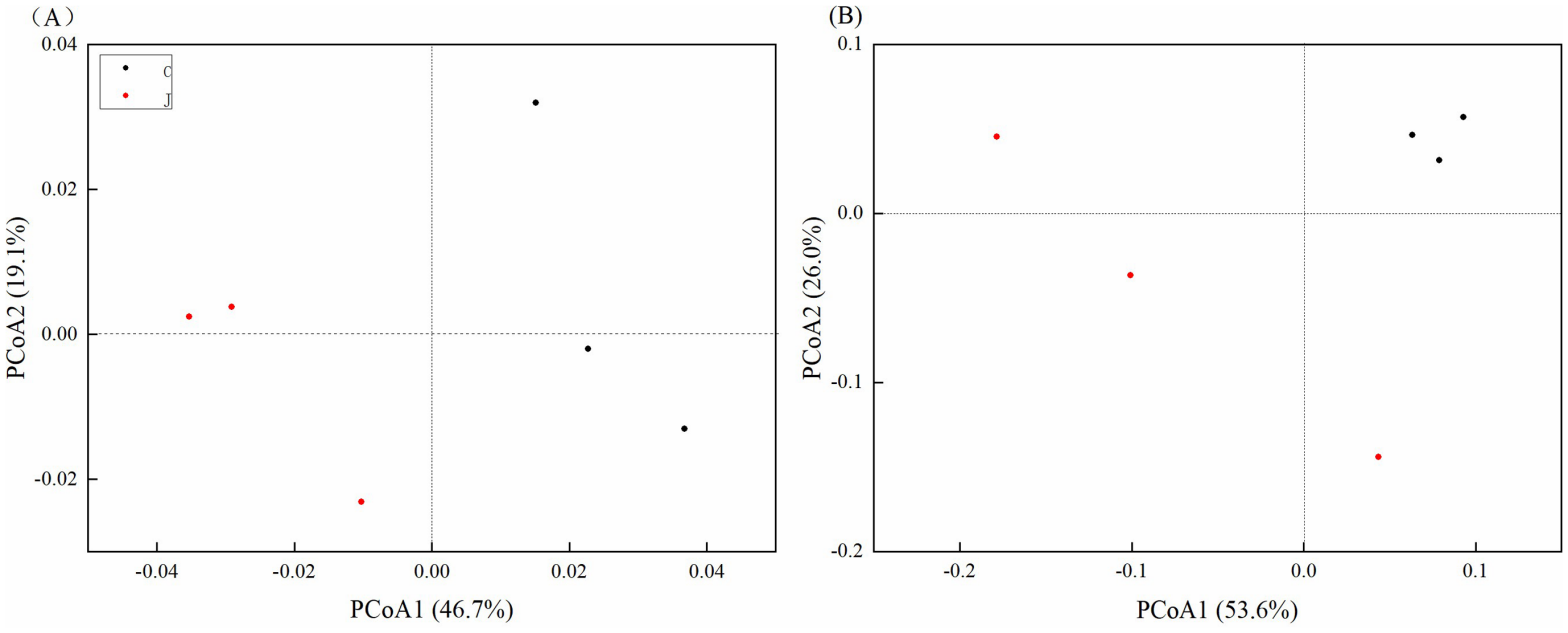
PCoA of soil bacterial community **(A)** and fungal community **(B)**.

Principal Coordinate Analysis (PCoA) provides a visual representation of differences in microbial community structure between samples, where closer sample distances indicate greater similarity in species composition. PCoA based on the Unifrac distance matrix revealed that for bacterial communities, PCoA1 and PCoA2 accounted for 46.7 and 19.1% of the sample variation, respectively, with a cumulative contribution rate of 65.8%. In fungal communities, PCoA1 and PCoA2 explained 53.6 and 26.0% of the variation, respectively, with a cumulative contribution rate of 79.6%, representing the primary source of differentiation. As illustrated in Figure 1A (bacterial communities), samples J (healthy plants) were distributed in the negative region of PCoA1 and both positive/negative regions of PCoA2, while samples C (red root rot-infected plants) clustered in the positive region of PCoA1 and both positive/negative regions of PCoA2, with relatively small separation distances between groups. In Figure 1B (fungal communities), samples J were scattered across both positive and negative regions of PCoA1 and PCoA2, whereas samples C aggregated distinctly in the positive quadrant of both PCoA1 and PCoA2. Notably, the three biological replicates of C formed a tight cluster in the first quadrant, demonstrating significant spatial separation from J samples. This clear partitioning indicates substantial differences in principal coordinates between healthy and diseased plant microbiomes.

ANOSIM and Adonis analyses of bacterial and fungal communities between healthy and diseased plants revealed no significant differences in overall composition (Table 3). However, the *p*-value for fungal community composition was smaller than that for bacterial communities, suggesting that fungal community structure may play a more important role than bacterial communities in disease development.

**Table 3:** Comparative analysis of soil microbial community composition in healthy and red root rot plants of rubber trees.

Classification	ANOSIM		*R* ^2^	Adonis	
	R	P		F	P
Bacteria	0.52	0.21	0.45	3.25	0.21
Fungus	0.81	0.11	0.86	23.93	0.11

### 3.4 Soil microbial community composition

**Figure 2:**
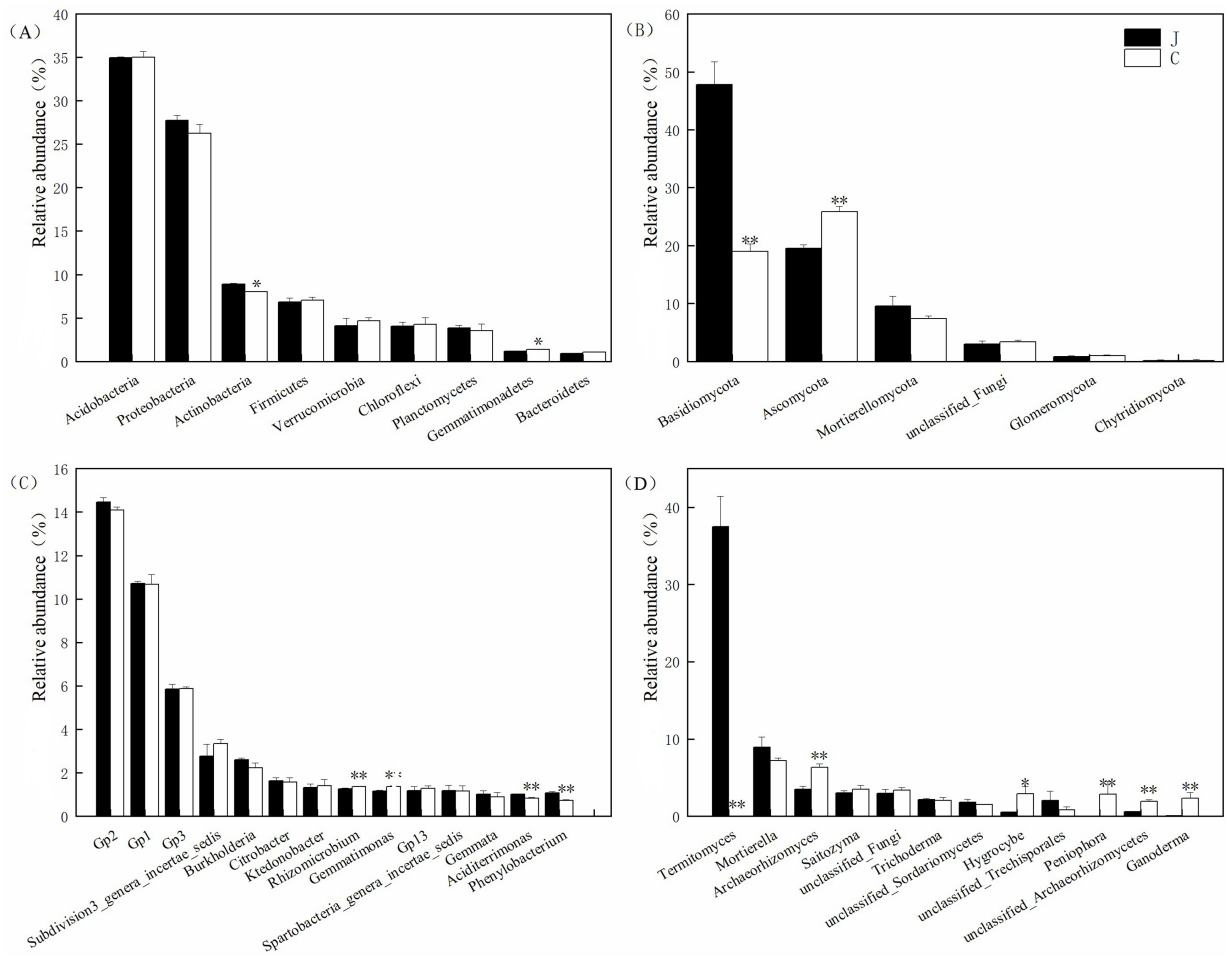
Taxonomic summary of the relative abundance of the root-zone bacterial phyla **(A)**, fungal phyla **(B)**, bacterial genera **(C)**, and fungal genus **(D)** in healthy and diseased soil. *p < 0.05, **p < 0.01.

Microbial community composition analysis of root-zone soils from healthy and diseased plants of rubber trees at phylum and genus levels. At the phylum level, the dominant bacterial phyla (relative abundance >5%) included Acidobacteria, Proteobacteria, Actinobacteria, and Firmicutes (Figure 2A). Compared with healthy soils, diseased soils showed a significant decrease (0.92%) in Actinobacteria and a significant increase (0.21%) in Gemmatimonadetes, while no significant differences were observed in other bacterial phyla (Figure 2A). For fungal communities, the predominant phyla (relative abundance >5%) were Basidiomycota, Ascomycota, and Mortierellomycota (Figure 2B). Notably, diseased soils exhibited a sharp decline (28.89%) in Basidiomycota and a significant increase (6.28%) in Ascomycota, with no significant variations in other fungal phyla between healthy and diseased soils (Figure 2B).

At the genus level, dominant taxa were defined as those with an average relative abundance >5%. In the bacterial communities of rubber tree root-zone soils, *Acidobacteria* Gp2, Gp1, and Gp3 were identified as the dominant genera in both healthy and diseased soils. Notably, the relative abundances of *Rhizomicrobium* and *Gemmatimonas* were significantly higher in diseased soils (*p* < 0.05), with increases of 0.10 and 0.21%, respectively. Conversely, *Aciditerrimonas* and *Phenylobacterium* showed significantly lower abundances in diseased soils (*p* < 0.05), decreasing by 0.19 and 0.38%, respectively (Figure 2C). No significant differences were observed for other bacterial genera. For fungal communities, *Termitomyces*, *Mortierella*, and *Archaeorhizomyces* were the dominant genera in both healthy and diseased soils. Strikingly, *Termitomyces* accounted for 37.50% of the fungal community in healthy soils but only 0.026% in diseased soils, while *Archaeorhizomyces* exhibited a lower relative abundance in healthy soils (3.48%) compared to diseased soils (6.34%). Additionally, the genera *Hygrocybe*, *Peniophora*, unclassified *Archaeorhizomycetes*, and *Ganoderma* showed markedly higher abundances in diseased soils, with increases of 82.13, 98.59, 69.74, and 97.87%, respectively (Figure 2D). The fungal community exhibited a dramatic decline in the symbiont *Termitomyces* (from 37.5 to 0.026%), alongside enrichment of saprotrophic genera (e.g., *Ganoderma*; +97.87%), suggesting a shift from mutualistic to pathogenic taxa in diseased soils. These genera contribute to the observed shifts in fungal community structure and may be associated with the development of red root rot.

### 3.5 Associations between soil microbial communities and soil physicochemical/enzymatic properties

Genus level correlations between soil microbial communities and physicochemical properties are summarized in Table 4. In the rubber tree root-zone bacterial communities, Acidobacteria subgroup *Gp2* showed a significant positive correlation with NH₄^+^-N (*p* < 0.05), while the seven other most abundant bacterial genera exhibited no significant associations with any soil properties (Supplementary Table S1). Among the dominant fungal genera, *Termitomyces* sp. was positively correlated with NO₃-N (*p* < 0.05) and NH₄^+^-N (*p* < 0.05) but negative correlated with pH (*p* < 0.01), TN (*p* < 0.01), AP (*p* < 0.01), SWC (*p* < 0.05), and SOM (*p* < 0.05). *Archaeorhizomyces* sp. displayed positively correlations with pH (*p* < 0.01), SWC (*p* < 0.01), TN (*p* < 0.01) and AP (*p* < 0.01), while showing a negative correlated with NO₃-N (*p* < 0.05). *Hygrocybe* sp. exhibited positively correlated with pH (*p* < 0.05), TN (*p* < 0.05), and AP (*p* < 0.05), but a negative correlation with NH₄^+^-N (*p* < 0.05). *Ganoderma* sp. was positively correlated with SWC (*p* < 0.01), AP (*p* < 0.01), pH (*p* < 0.05), and TN (*p* < 0.05).

**Table 4:** Correlations (Pearson’s r) between soil properties and dominant microbial genera.

Genera	pH	SWC	TN	NO_3_-N	NH4^+^-N	AP	AK	SOM
*Gp2*	−0.75	−0.46	−0.72	0.61	0.84*	−0.69	0.01	−0.69
*Gp1*	0.02	−0.20	−0.09	0.12	−0.05	−0.14	−0.07	0.00
*Gp3*	0.06	0.13	0.03	0.40	−0.09	0.06	0.35	0.12
*Subdivision3_genera*	0.55	0.54	0.58	−0.28	−0.73	0.67	−0.01	0.36
*Burkholderia*	−0.70	−0.67	−0.78	0.59	0.74	−0.80	−0.24	−0.74
*Citrobacter*	−0.07	−0.35	−0.14	−0.07	0.17	−0.28	−0.08	0.20
*Ktedonobacter*	0.21	−0.04	0.24	0.03	−0.33	0.18	−0.15	0.67
*Rhizomicrobium*	0.95**	0.76	0.92**	−0.79	−0.86*	0.86*	0.53	0.88*
*Gemmatimonas*	0.97**	0.84*	0.97**	−0.92**	−0.89*	0.94**	0.50	0.82*
*Gp13*	0.36	0.04	0.31	−0.48	−0.39	0.24	−0.22	0.35
*Spartobacteria_genera*	−0.06	0.05	−0.01	0.21	−0.16	0.12	−0.34	−0.22
*Gemmata*	−0.44	−0.19	−0.35	0.11	0.53	−0.33	−0.07	−0.41
*Aciditerrimonas*	−0.95**	−0.79	−0.97**	0.88*	0.89*	−0.92**	−0.44	−0.92**
*Phenylobacterium*	−0.97**	−0.88*	−0.98**	0.74	0.93**	−0.97**	−0.56	−0.88*
*WPS-2_genera*	−0.27	−0.58	−0.34	0.49	0.09	−0.39	−0.52	0.02
*Stella*	−0.85*	−0.86*	−0.90*	0.81	0.75	−0.89**	−0.56	−0.76
*Aquisphaera*	−0.42	−0.17	−0.34	−0.02	0.56	−0.33	−0.02	−0.44
*Labilithrix*	0.40	0.35	0.34	0.11	−0.55	0.41	0.17	0.21
*Bradyrhizobium*	0.80	0.70	0.80	−0.74	−0.64	0.72	0.65	0.82*
*Termitomyces*	−0.99**	−0.90*	−0.99**	0.91*	0.88*	−0.96**	−0.60	−0.81*
*Mortierella*	−0.66	−0.72	−0.68	0.24	0.78	−0.77	−0.35	−0.47
*Archaeorhizomyces*	0.95**	0.96**	0.95**	−0.86*	−0.79	0.93**	0.78	0.69
*Saitozyma*	0.56	0.46	0.48	−0.45	−0.37	0.40	0.65	0.41
*Trichoderma*	−0.12	−0.50	−0.10	0.23	−0.16	−0.15	−0.80	0.38
*Sordariomycetes*	−0.43	−0.55	−0.42	0.33	0.20	−0.38	−0.83*	−0.36
*Hygrocybe*	0.88*	0.63	0.89*	−0.72	−0.90*	0.84*	0.27	0.99
*Trechisporales*	−0.55	−0.64	−0.58	0.65	0.31	−0.53	−0.74	−0.51
*Peniophora*	0.75	0.94**	0.75	−0.68	−0.58	0.80	0.79	0.28
*unclassified Archaeorhizomycetes*	0.98**	0.84*	0.99**	−0.81	−0.96**	0.97**	0.44	0.91*
*Ganoderma*	0.89*	0.99**	0.89*	−0.80	−0.76	0.92**	0.75	0.52
*unclassified_Hypocreales*	−0.23	−0.52	−0.23	0.23	−0.05	−0.23	−0.89*	0.01
*unclassified_Xylariales*	−0.32	−0.38	−0.37	−0.10	0.54	−0.48	0.01*	−0.30
*unclassified_Nectriaceae*	−0.87*	−0.81*	−0.87*	0.90*	0.67	−0.81	−0.71	−0.75
*unclassified_Venturiales*	−0.51	−0.52	−0.44	0.25	0.34	−0.40	−0.78	−0.36
*Cladophialophora*	0.73	0.54	0.77	−0.85*	−0.65	0.69	0.24	0.85*
*unclassified_Glomeromycota*	−0.58	−0.68	−0.57	0.31	0.43	−0.56	−0.83*	−0.48

*p < 0.05, **p < 0.01.

Table 5 shows the Pearson correlation analysis between soil enzyme activities and soil microbial communities at the genus level. Among the dominant bacterial genera in the root-zone soil of rubber trees, *Gp2* (Acidobacteria) showed a significant positively correlated with URE (*p* < 0.05). *Rhizomicrobium* sp. exhibited positively correlated with CAT (*p* < 0.05) and INV (*p* < 0.05), but a significant negative correlated with ACP (*p* < 0.01). For the dominant fungal genera, *Termitomyces* sp. was positively correlated with ACP (*p* < 0.05) but negative correlated with CAT (*p* < 0.05) and INV (*p* < 0.01). In contrast, *Archaeorhizomyces* sp. displayed positively correlated with INV (*p* < 0.01) and CAT (*p* < 0.05), while showing a negative correlated with ACP (*p* < 0.05). Additionally, *Ganoderma* sp. demonstrated highly significant positively correlations with both CAT (*p* < 0.01) and INV (*p* < 0.01) (Supplementary Table S2).

**Table 5:** Correlations (Pearson’s r) between soil enzyme activities and dominant microbial genera.

Genera	URE	CAT	INV	ACP	PPO
*Gp2*	0.84*	−0.78	−0.66	0.60	0.12
*Gp1*	0.12	0.06	−0.02	−0.20	0.35
*Gp3*	0.45	0.02	0.17	−0.03	−0.45
*Subdivision3_genera*	−0.64	0.74	0.62	−0.03	−0.74
*Burkholderia*	0.73	−0.62	−0.68	0.47	0.53
*Citrobacter*	0.10	−0.30	−0.25	−0.46	0.72
*Ktedonobacter*	−0.17	−0.03	0.12	−0.38	−0.33
*Rhizomicrobium*	−0.71	0.82*	0.87*	−0.95**	0.02
*Gemmatimonas*	−0.87*	0.90*	0.91*	−0.86*	−0.03
*Gp13*	−0.60	0.37	0.21	−0.46	0.40
*Spartobacteria_genera*	−0.18	0.22	0.07	0.57	−0.70
*Gemmata*	0.23	−0.49	−0.42	0.44	0.05
*Aciditerrimonas*	0.86*	−0.81*	−0.87*	0.89*	0.11
*Phenylobacterium*	0.73	−0.87*	−0.95**	0.79	0.34
*WPS-2_genera*	0.23	−0.28	−0.33	0.05	0.06
*Stella*	0.72	−0.72	−0.82*	0.70	0.23
*Aquisphaera*	0.19	−0.46	−0.42	0.35	0.26
*Labilithrix*	−0.10	0.58	0.52	−0.07	−0.56
*Bradyrhizobium*	−0.50	0.57	0.72	−0.94**	0.11
*Termitomyces*	0.81	−0.91*	−0.94**	0.86*	0.07
*Mortierella*	0.43	−0.78	−0.79	0.20	0.82*
*Archaeorhizomyces*	−0.65	0.89*	0.95**	−0.82*	−0.04
*Saitozyma*	−0.12	0.45	0.52	−0.76	0.41
*Trichoderma*	−0.26	−0.20	−0.25	−0.01	−0.23
*unclassified_Sordariomycetes*	−0.10	−0.23	−0.45	0.57	−0.10
*Hygrocybe*	−0.82*	0.72	0.78	−0.87*	−0.18
*unclassified_Trechisporales*	0.27	−0.32	−0.51	0.68	−0.18
*Peniophora*	−0.45	0.83*	0.84*	−0.42	−0.15
*unclassified_Archaeorhizomycetes*	−0.86*	0.90*	0.93**	−0.80	−0.28
*Ganoderma*	−0.62	0.92**	0.94**	−0.61	−0.17
*unclassified_Basidiomycota*	−0.81	0.55	0.60	−0.53	−0.37
*unclassified_Hypocreales*	−0.28	−0.12	−0.32	0.27	−0.14
*unclassified_Xylariales*	0.27	−0.45	−0.45	−0.15	0.99**
*unclassified_Nectriaceae*	0.62	−0.69	−0.80	0.93**	−0.19
*unclassified_Venturiales*	−0.09	−0.41	−0.55	0.61	−0.11
*Cladophialophora*	−0.82*	0.51	0.58	−0.81*	0.09
*unclassified_Glomeromycota*	0.01	−0.45	−0.64	0.57	0.18

*p < 0.05, **p < 0.01.

## 4 Discussion

Soil microorganisms play a crucial role in nutrient cycling and fertility maintenance, serving as vital components of soil micro-ecological environments (Zhao et al., 2022; Kong et al., 2024). The abundance and community structure of soil microorganisms interact reciprocally with plant health status. Microbial population richness and variability significantly influence soil quality, functionality, and the sustainable development of soil ecosystems (Kennedy and Smith, 1995). Therefore, investigating the community structure and diversity of rhizosphere soil microorganisms in rubber trees affected by root rot represents a critical step in disease control and prevention. Generally, higher alpha diversity indices indicate more complex microbial community structures and greater stability (Gao and He, 2010). Multiple studies have revealed varying patterns of microbial diversity changes in plants affected by root rot. Jia et al. (2021) observed significantly lower *α*-diversity of fungal communities in rhizosphere soil of root rot of *Lycium barbarum* Ningqi-1 compared to healthy plants. Yang et al. (2020) reported higher fungal diversity indices and richness in apple root rot on rhizosphere soils versus healthy controls. Liu et al. (2023) found increased fungal α-diversity but decreased bacterial α-diversity in root rot of *Corydalis tomentella* rhizosphere soil relative to healthy plants. Li et al. (2025) demonstrated significantly enhanced bacterial α-diversity (both richness and evenness) in root rot of *Lonicera japonica*, while fungal α-diversity remained unchanged. Qin et al. (2023) found that Huanglongbing-infected Gannan navel orange (*Citrus sinensis*) rhizosphere soils in Jiangxi Province exhibited increased bacterial α-diversity (richness and Shannon index) but reduced fungal richness compared to healthy plants. Liu J. et al. (2022) revealed significantly greater diversity and abundance of both fungal and bacterial communities in healthy *Atractylodes lancea* rhizosphere soils than in root rot plants. In our study, both healthy and red root rot infected rubber trees exhibited significantly higher bacterial OTUs than fungal OTUs in root-zone soils (*p* < 0.01), indicating predominant bacterial colonization. These findings align with previous reports on crucifer clubroot disease (Wu et al., 2020) and *Lycium ruthenicum* rhizosphere/bulk soils (Li et al., 2018). There are significant differences in the number of root-zone microorganisms. The species, population diversity, and richness of fungi and bacteria in the rhizosphere soil of rubber plants infected with red root rot are significantly higher than those of healthy plants. This result is consistent with the research findings on microbial communities related to cucumber fusarium wilt (Miao et al., 2004), apple root rot (Yang et al., 2020), *Panax notoginseng* root rot (Wu et al., 2016), and *Knoxia roxburghii* root rot (Liu et al., 2024). However, it is not entirely consistent with the research results on *Corydalis tomentella* root rot (Liu et al., 2023), *Lonicera japonica* root rot (Li et al., 2025), and *Hevea brasiliensis* red root rot in Changjiang County, Hainan Province (Tu et al., 2021). These discrepancies may be related to differences in the metabolic capacity and adaptability to biological stress among plants.

Microorganisms exhibit highly sensitive to soil environmental conditions. Multiple factors including soil nutrient status, agricultural practices, plant species, and climate variations can significantly modify soil microbial community structure and composition (Liu et al., 2020; Deng C. C. et al., 2019; Deng J. J. et al., 2019). The onset of soil-borne plant diseases induces distinct alterations in rhizosphere microbial profiles and dominant populations (Laurent et al., 2010; Aranda et al., 2011). Empirical evidence demonstrates disease-specific microbial shifts. Naked barley (*Hordeum vulgare*) root rot causes depletion of beneficial bacterial taxa, particularly Actinobacteria (Li et al., 2017a). *Coptis chinensis* rhizosphere shows marked proliferation of Proteobacteria following root rot infection (Tang et al., 2022). Apple root rot results in significantly reduced Ascomycota abundance compared to healthy controls (Yang et al., 2020). Japanese honeysuckle (*Lonicera japonica*) maintains similar overall rhizosphere composition during root rot infection but exhibits elevated Proteobacteria and Actinobacteria abundances (Li et al., 2025). In our study, red root rot significantly altered microbial community structure in the root-zone soil, with substantial shifts in the abundance of dominant fungal and bacterial populations. Notably, the composition of fungal communities exerted a more pronounced influence on disease progression compared to bacterial communities. The most abundant fungal phyla in the rhizosphere soil were Basidiomycota, Ascomycota, and Mortierellomycota, while the dominant bacterial phyla included Acidobacteria, Proteobacteria, Actinobacteria, and Firmicutes. Relative to healthy samples, plants infected with red root rot exhitited significantly lower relative abundances of Actinobacteria and Basidiomycota, alongside a significantly higher relative abundance of Ascomycota. These findings are consistent with previous research on root rot in *Atractylodes chinensis* (Gao et al., 2022), *Polygonatum sibiricum* (Lu et al., 2021), crucifer clubroot (Wu et al., 2020), and *Paeonia lactiflora* (Zhang et al., 2024).

Previous studies have shown that Acidobacteria are oligotrophic bacteria (Johnston-monje et al., 2016) capable of surviving in environments with scarce soil nutrients (Li et al., 2020). They can decompose plant residue polymers, participate in iron cycling and one-carbon compound metabolism, and thus play an important role in soil ecosystems (Wang et al., 2016). Proteobacteria have the functions of decomposing organic matter, nitrates and ammonium salts, and promoting plants’ absorption and utilization of nitrogen (Berlemont and Martiny, 2013). Actinobacteria can produce a variety of antibiotics, which play a significant role in regulating the ecological balance of soil microorganisms (Qin et al., 2023). Most of them are saprophytes, which can accelerate the decomposition of animal and plant residues in rhizosphere soil and promote the material cycle of nitrogen (Tang et al., 2024). Similarly, rhizosphere fungi, as an important component of the rhizosphere ecosystem, also play a crucial role in maintaining the stability of the rhizosphere ecosystem, protecting plants from pathogenic microorganism, and promoting plant growth and development (Tedersoo et al., 2014). As the main soil fungal decomposers, Ascomycota and Basidiomycota play a significant role in degrading organic matter in rhizosphere soil and improving soil fertility (Bastian et al., 2009). At the genus level, the dominant bacterial genus in the root-zone soil bacterial community in this study was *Acidobacterium*, while *Termitomyces*, *Mortierella*, and *Archaeorhizomyces* were the dominant genera in the root-zone soil fungal community, with *Termitomyces* showing an extremely high relative abundance in the soil fungal community of healthy plant. *Termitomyces* is an obligate symbiotic fungus of termites, capable of secreting cellulase and laccase to promote organic matter mineralization (Hyodo et al., 2003). In addition, compared with healthy rubber trees, the abundances of *Hygrocybe*, *Peniophora*, unclassified *Archaeorhizomycetes*, and *Ganoderma* in the root-zone soil of red root rot-infected plants increased significantly, by 82.13, 98.59, 69.74, and 97.87%, respectively, compared to those in the soil of healthy plants. *Ganoderma* sp. can infect plants, leading to wilting and root rot, and is a pathogenic fungus responsible for red root rot in rubber trees (Liang et al., 2022). This study found that the abundance of *Ganoderma* was extremely high in the root-zone soil of infected rubber trees, making it a key genus driving changes in the fungal community composition. It is speculated that *Ganoderma* may be the main microbial group causing the occurrence of red root rot in local rubber trees.

Soil physicochemical properties are closely linked to enzyme activities and microbial dynamics in the rhizosphere of plants. Soil enzymes mediate critical biochemical processes and play a vital role in nutrient transformation (Zhou, 1987). Key enzymes such as URE, CAT, PPO, ACP, and INV are essential for the cycling of nutrients, carbon, and phosphorus, as well as plant defense mechanisms (Abay et al., 2022). In our study, the rhizosphere soil of rubber trees infected with red root rot exhibited significantly higher pH, soil water content (SWC), total nitrogen (TN), and activities of INV and CAT compared to healthy plants. Conversely, NO₃-N content and ACP activity were significantly lower in diseased soils. These findings suggest that red root rot may impair the root and microbial capacity for total nitrogen and water uptake while accelerating NO₃-N metabolism. The reduced ACP activity further indicates that the disease disrupts soil nutrient cycling, aligning with prior studies (You et al., 2014; Xun et al., 2013; Li et al., 2017b). The elevated pH in diseased soils creates a weakly acidic environment, which likely facilitates the growth, infection, and spread of red root rot pathogens. This observation supports earlier research (He et al., 2019) but contrary with findings by Tu et al. (2021). Soil pH plays a critical role in determining soil physicochemical properties and serves as a key driver of rhizosphere microbial community composition (Dhaigude et al., 2024). Huang and Liu (1993) examined the correlation between red root rot incidence in rubber trees and soil pH in rubber plantations in Yunnan Province, finding no consistent pH variation patterns across different infected forest sections. Zhang Y. Y. et al. (2023) demonstrated that pH, electrical conductivity (EC), NO₃-N, AP, and AK are primary determinants of microbial community shifts in continuous cropping systems of medicinal plants. Similarly, Zhang Z. H. et al. (2023) established that pH, TN, alkali-hydrolyzable nitrogen (AHN), available potassium (AK), and cation exchange capacity significantly affected bacterial and fungal communities in both healthy and clubroot-infected Chinese cabbage. In the rhizosphere soil of *Coptis chinensis* plants with root rot, AK, available nitrogen (AN), and INV activity are significantly higher than those in healthy plants, while CAT activity shows significant reduction (Song et al., 2017). In the rhizosphere soils of diseased *Atractylodes lancea* exhibit substantially higher levels of SOM, AN, AP, AK, and increased URE and ACP activities compared to healthy plants (Liu J. et al., 2022). The INV activity in the rhizosphere soil of *Lonicera japonica* with root rot was significantly reduced, which is a key environmental factor affecting their rhizosphere microbial structure (Li et al., 2025). This study found that the correlation analysis between rhizosphere microorganisms, soil physicochemical properties, and enzyme activities revealed that soil pH, TN, SWC, AP, CAT, and INV were positively correlated with the relative abundance of *Ganoderma* and *Archaeorhizomyces*, while NO₃-N content and ACP showed positive correlations with the relative abundance of *Termitomyces*. These research results indicate that soil conditions vary, and different crops have distinct effects on soil physicochemical properties and enzyme activities. Therefore, the disease resistance of rubber trees can be improved by reducing soil pH, decreasing nitrogen fertilizer, and increasing phosphorus fertilizer application. Finally, the interaction between the rhizosphere soil microbial community and soil physicochemical properties in rubber trees in Qiongzhong, Hainan, influences the occurrence of red root rot. This interaction and its underlying mechanisms warrant further in-depth study, which will provide new insights into understanding the rhizosphere soil microecological mechanisms of rubber tree red root rot and its effective prevention and control.

## 5 Conclusion

In this study, high-throughput sequencing technology was used to investigate the microbial community structure in the rhizosphere soil of healthy rubber trees and those infected with red root rot. After the infection that caused red root rot, there was no significant difference in the bacterial and fungal community structures between the rhizosphere soil of healthy plants and that of diseased plants, but the composition of the fungal community played a greater role in affecting plant pathogenesis than the bacterial community. The occurrence of red root disease affected the diversity, structure, and composition of fungal and bacterial communities in the soil. The richness and *α*-diversity of bacterial and fungal populations in the rhizosphere soil increased. The abundance of Actinobacteria in the root zone of diseased plants was significantly lower than that of healthy plants, while the abundance of Ascomycota was significantly higher. In the soil of diseased plants, the fungal community shifted from mutualistic groups to pathogenic groups. Changes in the abundance of *Termitomyces*, *Hygrocybe*, *Peniophora*, unclassified *Archaeorhizomycetes*, and *Ganoderma* may be the main causes of red root rot in rubber trees. There was a certain correlation between soil environmental factors and the root-zone microbial community structure of rubber trees such as *Ganoderma*, *Archaeorhizomyce*. Further in-depth research on their interaction relationships and mechanisms is needed to improve the stability and disease resistance of the rhizosphere microbial community structure of rubber trees, thereby providing a theoretical basis for the regulation of the soil microecology of rubber trees.

## Data Availability

The sequencing datasets of bacterial 16S rRNA and fungal ITS genes in high-throughput sequencing to the NCBI SRA database. The BioProject accession number is PRJNA1440924 (URL: https://www.ncbi.nlm.nih.gov/bioproject/PRJNA1440924).
